# Auscultation and Percussion

**Published:** 1854-10

**Authors:** 


### BIBLIOGRAPHICAL NOTICES.

*Auscultation and Percussion.* By JOSEPH SKODA. *Translated from the Fourth German Edition.* By W. O. MARKHAM, M. D., Assistant Physician to St. Mary's Hospital. Philadelphia, Lindsay & Blakiston, 1854.

*A Practical Treatise on the Diseases of the Lungs, Heart and Aorta, including the principles of Physical Diagnosis.* By WALTER HAYLE WALSHE, M. D., Prof. of Clinical Medicine in University College, London. *Second Edition.* London, Walton & Maberly, 1854.

*A Clinical Introduction to the practice of Auscultation and other modes of Physical Diagnosis in diseases of the Lungs and Heart.* By H. M. HUGHES, M.D. *Second American from the Second English Edition.* Philadelphia, Blanchard & Lea, 1854.

The advantages derived from an accurate study of physical diagnosis cannot be too highly appreciated, for notwithstanding the continued labors of so many of the older physicians, we may safely say, that all exact knowledge of diseases, involving lesions of the internal organs, and especially those of the thorax, dates from the introduction of this method of investigation. We cannot, therefore, be too thankful to the genius and application of those men, who not only discovered, but almost completed, a system which places us so inestimably in advance of our medical forefathers; and which, by making medicine more and more a science, based on correct observation, gave to its cultivation an impetus, the beneficial effects of which will be felt for centuries to come.

As, however, with all new discoveries, so was it with the introduction of Percussion and Auscultation into practice. At first violently contested, it became soon so popular a method as to cause many of its followers to rely on it exclusively, and to discard altogether the general symptoms furnished by disease. This over confidence in the physical signs, and an attempt at too great a refinement, produced, especially in Auscultation, a tendency to discover separate pathognomic signs for each thoracic affection. Laennec is, we think, himself to blame for this. Had this great man striven more to ascertain the exact lesions and physical causes, and not so much the diseases which produce the sounds he so admirably described, his influence on the progress of auscultation would have been more beneficial. His great authority retarded also from original investigation, many who felt that some of his views (and hence those at present taught,) needed reforming or confirming in several respects. It was to refute some of the observations and arguments of Laennec, as well as against the tendency of seeking pathognomic signs in auscultation, that Professor Skoda produced his admirable work on auscultation; a treatise embodying the profoundest thought with the closest observation, and one which, to use the language of his translator, Dr. Markham, "will modify many of the opinions of the present day, even should the theories offered in their place be rejected."

Dr. Skoda commences with percussion. Piorry and his pupil Mailliot, have led us to believe that the spleen, the kidney or hepatized lung yield differences in the percussion sound, by which they may be distinguished. This Skoda denies. He states, on the contrary, that the percussion sound emitted by organs not containing air, does not vary: and that even the sounds furnished by fluids, (water, pus or blood,) are in no way distinguishable from those of solids; both giving a dull sound.

The normal sounds obtained by percussion have been variously classed. The simplest division of the sounds is the one of Læennec, into clear and dull. Skoda distinguishes four varieties, the extremes of which he represents by the following terms:

1. Full—Empty.
2. Clear—Dull.
3. Tympanitic—Nontympanitic.
4. High—Low.

By a full sound, Skoda means a sound such as is normally heard in percussing the stomach, when distended with air. It characterizes the size of bodies, is persistent, and spread, as it were, over a large surface; an empty sound is the reverse of this. The term clear and dull are used in their ordinary signification. By the term tympanitic, Skoda understands the sound heard on percussing the abdomen. High and low sounds are only varieties of pitch, and are, as he himself admits, but of little value in practice.

The terms full and empty, are not employed by any other author; of their meaning, Skoda says:

"We do not judge of the size of a resonant body, by the strength of the sound which strikes upon the ear; the slightest vibration of a large bell, tells of its magnitude; the loudest ring of a little bell misleads no one, as to its smallness; neither do we judge of the dimensions of bodies from the pitch of their sounds.

There is no good general term to designate that quality of sounds, which characterizes the size of bodies. I believe that in singing and instrumental music, the word full or full toned or sonorous is used; I shall, therefore, borrow the expression, in speaking of the percussion sound. When any one percusses with equal force different parts of thorax and abdomen, he will find that in some places, the sound appears more persistent, and, as it were, spread over a larger surface than it does in others; the first kind of sound, I call the full; the second, the less full or empty percussion sound.

A cavity, superficially situated in the lungs of moderate size, and surrounded by thickened parenchyma, yields a very distinct percussion sound, but of an empty character. The stomach distended with air gives a full, the small intestines an empty sound." (p. 42.)

Now, we must confess, that we do not see any advantage to be derived from an introduction into the nomenclature of percussion of the terms *full* and *empty*. It is true, that a body containing much air, will yield a more persisting and more distinct sound; but to apply this law of acoustics to determine portions of diseased tissue, does not seem to us of much practical utility. Nor can we help believing that it would be a matter of no small difficulty to learn to separate these sounds; to distinguish, for instance, between a clear empty and clear full, or a dull full and a dull empty sound.

The *tympanitic* sound Skoda does not regard as dependant on quantity of air an organ contains, but rather on the amount of the tension of its parietes. If these be stretched, the sound ceases to be tympanitic and becomes dull; while the reverse is the case, if the walls be relaxed. The correctness of this statement may be readily proved by a simple experiment, viz: by percussing a stomach, which is being gradually more and more inflated with air. The more it becomes distended, the duller will the percussion-sound be.

The production of a tympanitic sound, by the relaxation of the walls of an organ containing air, is sometimes seen in the lungs.

"If the lung contains less than its normal quantity of air, it yields a sound which approaches to the tympanitic, or is distinctly tympanitic. The sound is, moreover, in many cases remarkably tympanitic, even when the diminution of the quantity of air in the lung is the effect of an increase in its fluid or solid constituents; and this, too, whether the lung retains its normal volume, or becomes larger than natural. When the lung is much reduced in volume by compression, but still contains air, its sound is invariably tympanitic." (p. 47.)

This theory of the production of the tympanitic sound, by diminution of the air in the lungs, Skoda further applies to the explanation of the singular phenomenon first described by him, that if the lower portion of a lung be compressed by a pleuritic effusion, the percussion-sound at the upper portion of the thorax becomes distinctly tympanitic. The same phenomenon may be observed at the apex of the lung in infiltrated tubercle. A case came lately under the notice of the reviewer, where this existed to a marked degree. Can its production here also be referred to the same cause? We see no objection to this view.

As abnormal sounds, yielded by percussion, Skoda classes the cracked pot sound, and the metallic sound. For the production of the latter sound (on percussion as well as on auscultation,) he does not regard the presence of fluid as indispensable. He denies, also, the "hydatid" sound of Piorry as a distinct sound; its singularity, he conceives to arise from a peculiar vibration communicated to the finger, while percussing.

The subject of Auscultation is the one next discussed by Skoda. To enter minutely into a critical examination of all the novel doctrines here advanced, would prolong the review far beyond the limits assigned to it. We will, therefore, merely note those points which we deem of more essential interest, and endeavor as we proceed to show, in what the difference between the views of Skoda and of Laennec and his followers consist.

The *auscultation of the voice* is evidently a subject to which Skoda has paid great attention. Naturally, when listening to the thorax the voice is heard as an indistinct humming sound. When from any cause the tissue of the lung becomes denser, either by consolidation or by infiltration, the result of morbid processes, the voice, instead of being heard as a mere humming, becomes distinct, giving rise to bronchophony or the distinct thoracic voice. This increase of distinctness was attributed by Laennec to the increase of the conducting power of the lung; and this explanation has been received into most of the English and French manuals on Auscultation, with which we are acquainted. But this hypothesis fails to account for all the circumstances observable in the study of bronchophony. Thus, bronchophony is not always a stationary sign, as in the course of a few minutes whilst listening over hepatized lungs it may appear and disappear, without any corresponding alteration, discoverable by percussion or otherwise, being observable in the part. It is further continually observed in pleurisy, that the thoracic voice becomes weaker, as the effusion advances, the reverse of which ought to happen in accordance with the ordinary laws of the conduction of sound. Skoda opposes, however, Laennec's explanations mainly on physical grounds and especially on the ground that sounds are better propagated in the air than by solids. But let us quote his own argument, as rendered by Markham:

"The human voice, and every other sound which is formed or propagated in the air, is heard very indistinctly, or not at all, by a person under water; and a sound in one room passes with difficulty into another, being interrupted by the walls. Any one wishing to weaken his hearing, stops his ears.

On the other hand, the slightest scratching at one end of a long rod may be heard, if the ear be brought in contact with the other: while no sound whatever is audible in the air, although the ear be brought much nearer to that end of the rod whence the sound proceeds. The sound caused by striking two stones together, under water, is distinctly heard there, and even causes a disagreeable sensation; whilst, out of water, it can be scarcely recognized. These facts show that sound does not pass readily from dense bodies into the air, or from the air into dense bodies. Physics also, teach us that sound is always reflected, in passing from one medium into another, and that less sound enters into the new medium than would have been propagated through a corresponding space of the one in which it was originally excited; the more dissimilar the media are, in respect of density and cohesion, the greater is the reflection of the sound, and the less freely does it pass from the one into the other."

Now according to these laws it would certainly follow that the healthy lung is as good or even a better conductor of sound than a condensed lung (since it allows the air or the sound to pass uninterruptedly into the air-cells,) and hence that increased thoracic voice cannot depend upon hepatized lung being a better sound-conductor.

As the conduction of sound fails to account for the phenomenon of bronchophony, Skoda seeks another physical fact by which it may be explained. It is a well known law of acoustics that vibrations are excited, and even increased in strength, in sounding bodies, when another body of the same pitch has been made to vibrate in its immediate neighborhood. On this principle the fact is explained, that a tuning fork in vibration will excite a reciprocal sound on a guitar string near it, which sound will be increased in strength, if the sounding column divide into a number of shorter parts, each of which will re-produce the original sound. The force of this reciprocation and resonance of sound or "consonance" depends, in an enclosed space, much upon the nature of the walls: a space bounded by solid walls reflecting the sound strongly, whilst one bordered by thin walls will scarcely, if at all, increase the strength of the sound. On this principle Skoda has endeavored to explain the production of the thoracic voice. Naturally, he thinks, the parenchyma of the lung, being neither firm nor tense in structure, is unable to consonate with the voice formed in the larynx or with the consonant sounds of the throat, and of the cavities of the mouth and nose. The air contained in the trachea and bronchial tubes consonates with the voice, in so far as the walls confining it have, in respect to their power of reflecting sound, an analogous condition to the walls of the larynx. As the bronchial tubes pass into the structure of the lung they become thinner and lose their cartilaginous walls. The consonance of the voice must hence decrease as the tubes change their firm structure. Now if by any process the walls of the tubes become increased in thickness, or the tissue of the lung around them condensed and deprived of air, the necessary conditions for consonance with the sound produced in the larynx will be kept up, and the vibrations thus generated, will be reflected as distinct sounds, even through several inches of fleshy parts or fluids, to the surface of the thorax, provided of course the consonance between the air in the tubes and the air in the larynx remain uninterrupted.

Such is in the main Skoda's account of the production of the thoracic voice by consonance. That there are grounds on which this theory may be opposed, we are aware, but altogether it seems to us the most philosophical explanation of the production of the thoracic voice that has as yet been proposed. The principal objection that can, we think, be urged against Skoda's theory, is, that the laws of sound are as yet too imperfectly understood, to apply the principle of consonance so exclusively to the explanation of a vital phenomenon; but if this application be too general or not, future researches will show; in the meantime we must beg leave to refer our readers for an admirable summary of the objections to this theory to the critical examination of it in the chapter on voice in Prof. Walshe's work.

Laennec divided bronchophony according to its intensity and timbre into simple bronchophony, pectoriloquy and egophony. Skoda, to be consistent to his theory of consonance, admits only a weak and strong bronchophony. This nomenclature is certainly simple, yet its advantages seem to us doubtful, for it will be hard to fix the exact limits of weak bronchophony. The term strong bronchophony is further but another expression for pectoriloquy, since Laennec himself defines this simply as "the voice passing wholly through the stethescope," which he states to occur most frequently by the resonance of the voice in cavities. *Pectoriloquy* or strong bronchophony as a sign of a cavity is strongly condemned by Skoda, and indeed most auscultators now agree on this point. Cases are not of unfrequent occurrence, where a cavity existed without giving rise to pectoriloquy, or in which a post-mortem examination revealed the absence of an excavation, in a spot in which during life the most perfect pectoriloquy has been heard. Dr. Watson* relates an instance of this kind; Latham† and Stokes‡ attribute but little value to this sign: Walshe admits it as a distinct sound, but considers its significance unsettled.

*Egophony* was considered by Laennec as indicating a moderate pleuritic effusion. Skoda rejects it altogether as a sign of effusion, and affirms that it may be heard in hepatized or tuberculous lung, and normally in old women and children. That egophony may be produced in large as well as in a moderate effusion, was

* Lectures on the Practice of Physic Am. Ed. p. 635.

† Lectures on Clinical Medicine, Am. Ed. p. 112.

‡ Treatise on the Chest, Am. Ed. p. 378.

proved by a case published by Andral (Clin. Med. ii. Ob. xxi.); in which this sign was heard, even though the fluid in the pleura was so abundant as to have caused displacement of the heart. Many of the most distinguished of the immediate followers of Laennec have observed egophony in simple pneumonia, although Grisolle (De la Pneumonie), and lately Walshe, most strongly contend against the fact of its occurrence in this disease. Fournet regards egophony as only of real value as a sign of a pleuritic effusion, when it corresponds with other signs, either physical or rational, and such, indeed, is the view which is now generally taken of its significance.

The sounds caused by the motion of the air during respiration, are classed by Skoda under the head of respiratory murmurs, of râles, and of whistling and sonorous sounds. The character of the *respiratory murmurs* may be imitated by drawing air into and forcing it out of the mouth, whilst the lips are in the requisite position to pronounce a consonant and a vowel.

Thus the consonants which answer to the vesicular murmur will be, during inspiration, *v* and *b*; during expiration, between *f* and *h*. The murmurs of the larnyx, trachea and large bronchi, are represented by *ch*, which is combined with the vowel *e* to produce the highest, with the letter *u* for the lowest pitch.

"In attempting to imitate these murmurs, we find that in each case we retain the same consonant, and that whatever difference arises, depends upon a change of the vowel employed. The consonant which answers to these murmurs is *ch*, guttural, for it falls between *h* and *ch*. The laryngeal, tracheal, and bronchial murmurs, may be imitated by forcing air against the hard palate; this is done involuntarily in hard breathing. The pitch of the murmur depends upon the width of the opening through which the air passes, that is, the *ch* appears in combination with different vowels. As a rule, the laryngeal murmur is higher than the respiratory murmur of the lung."

The division of the respiratory murmur adopted by Skoda is very simple. He distinguishes,

1. The pulmonary respiratory murmur.
2. Bronchial breathing.
3. Amphoric echo, or metallic tinkling.
4. Indeterminate respiratory murmurs.

The *pulmonary respiratory* murmur, (the vesicular breathing of Andral), Skoda attributes, with Laennec, to the friction of the air against the walls of the air cells, the contractile power of which it overcomes. To prolonged expiration, which was considered by Jackson and Fournet as pathognomic of tubercular infiltration of the lung, he assigns but slight importance.

"I am, nevertheless, still of opinion that an increased expiratory murmur—provided it has not a bronchial nor any other character than that proper to it—indicates nothing more than this, that the air, in passing out of the lungs, meets with some obstruction in the bronchial tubes."

*Bronchial respiration* is accounted for by the laws of consonance, and offers the same indications as weak brochophony. Under the head of bronchial respiration Skoda includes Laennec's cavernous respiration, which he considers, like pectoriloquy, to be a sound of doubtful value, existing frequently without any signs of a cavity being found, and totally inadequate in itself to the diagnosis of an excavation. When the respiration becomes more amphoric, or is accompanied with metallic resonance, Skoda does not deny its being a sign of an excavation of the pulmonary tissue.

That bronchial respiration may occur when cavities exist, and cavernous breathing, on the other hand, be sometimes heard in solidified tissue, is a fact admitted by most observers. Yet on that account to strike from the nomenclature of auscultation a sound, which is so frequently met with, and which differs to the ear so entirely from the ordinary bronchial respiration, does not seem to us advisable. It were better, to avoid confusion, to curtail its significance, but retain the name.

Such respiratory murmurs, which give us no information as to the state of the parenchyma of the lung, Skoda denominates *indeterminate* sounds. These indeterminate sounds have neither the character of vesicular, nor of pure bronchial respiration; they include many of the sounds that we are accustomed to designate as varieties of the vesicular murmur. Now, although we know, that there are certain forms of the respiratory sounds found in so many dissimilar diseases of the lung, as to become of very slight, if of any, value to the diagnosis of the exact condition of the thoracic organs, the introduction of the term "indeterminate" may, in our humble opinion, be productive of great evil, and may much increase the difficulty of learning auscultation. Thus we venture to say that no two persons would be able to agree on the sound to be called "indeterminate." What to an ordinary auscultator might be an indeterminate sound, would not be apt to be such to the practiced ear of a Skoda, a Louis or a Walshe, whilst we think it not improbable that a beginner would consider all respiratory sounds as "indeterminate." DaC.

(To be continued.)

